# Supplementary material for: HIVprotI: an integrated web based platform for prediction and design of HIV proteins inhibitors
Source: J Cheminform. 2018 Mar 9;10:12. doi: 10.1186/s13321-018-0266-y (PMC5845081; doi:10.1186/s13321-018-0266-y)
Supplement: Supplementary file 2 — Additional file 2. Source code of HIVProtI web server. [file 13321_2018_266_MOESM2_ESM.zip › HIVprotI_Source-code/hivprotI/weka-3-6-6/doc/weka/core/mathematicalexpression/Scanner.html]

Scanner


---


|  |  |  |
| --- | --- | --- |
| **Overview** \| **Package** \| **Class** \| **Tree** \| **Deprecated** \| **Index** \| **Help** \| **Weka's home** | |  |
| **PREV CLASS**   **NEXT CLASS** | **FRAMES**    **NO FRAMES**     **All Classes** |  |
| SUMMARY: NESTED \| FIELD \| CONSTR \| METHOD | DETAIL: FIELD \| CONSTR \| METHOD |  |


---


## weka.core.mathematicalexpression Class Scanner

```
java.lang.Object
  weka.core.mathematicalexpression.Scanner
```

**All Implemented Interfaces:**: java\_cup.runtime.Scanner

---

``` public class Scanner extends java.lang.Object implements java_cup.runtime.Scanner ```

A scanner for mathematical expressions.

**Version:**
:   $Revision: 4942 $

**Author:**
:   FracPete (fracpete at waikato dot ac dot nz)

---

| **Field Summary** | |
| --- | --- |
| `static int` | `YYEOF`             This character denotes the end of file |
| `static int` | `YYINITIAL`             lexical states |


**Constructor Summary**

`Scanner(java.io.InputStream in)`
  
          Creates a new scanner.

`Scanner(java.io.InputStream r,
java_cup.runtime.SymbolFactory sf)`

`Scanner(java.io.Reader in)`
  
          Creates a new scanner
There is also a java.io.InputStream version of this constructor.


| **Method Summary** | |
| --- | --- |
| `java_cup.runtime.Symbol` | `next_token()`             Resumes scanning until the next regular expression is matched, the end of input is encountered or an I/O-Error occurs. |
| `void` | `yybegin(int newState)`             Enters a new lexical state |
| `char` | `yycharat(int pos)`             Returns the character at position pos from the matched text. |
| `void` | `yyclose()`             Closes the input stream. |
| `int` | `yylength()`             Returns the length of the matched text region. |
| `void` | `yypushback(int number)`             Pushes the specified amount of characters back into the input stream. |
| `void` | `yyreset(java.io.Reader reader)`             Resets the scanner to read from a new input stream. |
| `int` | `yystate()`             Returns the current lexical state. |
| `java.lang.String` | `yytext()`             Returns the text matched by the current regular expression. |

**Methods inherited from class java.lang.Object**

`equals, getClass, hashCode, notify, notifyAll, toString, wait, wait, wait`

**Field Detail**

### YYEOF

```
public static final int YYEOF
```

:   This character denotes the end of file

    **See Also:**: Constant Field Values

---


### YYINITIAL

```
public static final int YYINITIAL
```

:   lexical states

    **See Also:**: Constant Field Values


**Constructor Detail**

### Scanner

```
public Scanner(java.io.InputStream r,
               java_cup.runtime.SymbolFactory sf)
```

---


### Scanner

```
public Scanner(java.io.Reader in)
```

:   Creates a new scanner
    There is also a java.io.InputStream version of this constructor.

    **Parameters:**: `in` - the java.io.Reader to read input from.

---


### Scanner

```
public Scanner(java.io.InputStream in)
```

:   Creates a new scanner.
    There is also java.io.Reader version of this constructor.

    **Parameters:**: `in` - the java.io.Inputstream to read input from.


**Method Detail**

### yyclose

```
public final void yyclose()
                   throws java.io.IOException
```

:   Closes the input stream.

    :   **Throws:**: `java.io.IOException`

---


### yyreset

```
public final void yyreset(java.io.Reader reader)
```

:   Resets the scanner to read from a new input stream.
    Does not close the old reader.
    All internal variables are reset, the old input stream
    **cannot** be reused (internal buffer is discarded and lost).
    Lexical state is set to ZZ\_INITIAL.

    :   **Parameters:**: `reader` - the new input stream

---


### yystate

```
public final int yystate()
```

:   Returns the current lexical state.

---


### yybegin

```
public final void yybegin(int newState)
```

:   Enters a new lexical state

    :   **Parameters:**: `newState` - the new lexical state

---


### yytext

```
public final java.lang.String yytext()
```

:   Returns the text matched by the current regular expression.

---


### yycharat

```
public final char yycharat(int pos)
```

:   Returns the character at position pos from the
    matched text.
    It is equivalent to yytext().charAt(pos), but faster

    :   **Parameters:**: `pos` - the position of the character to fetch. A value from 0 to yylength()-1. **Returns:**: the character at position pos

---


### yylength

```
public final int yylength()
```

:   Returns the length of the matched text region.

---


### yypushback

```
public void yypushback(int number)
```

:   Pushes the specified amount of characters back into the input stream.
    They will be read again by then next call of the scanning method

    :   **Parameters:**: `number` - the number of characters to be read again. This number must not be greater than yylength()!

---


### next\_token

```
public java_cup.runtime.Symbol next_token()
                                   throws java.io.IOException
```

:   Resumes scanning until the next regular expression is matched,
    the end of input is encountered or an I/O-Error occurs.

    :   **Specified by:**: `next_token` in interface `java_cup.runtime.Scanner`
    :   **Returns:**: the next token **Throws:**: `java.io.IOException` - if any I/O-Error occurs


---


|  |  |  |
| --- | --- | --- |
| **Overview** \| **Package** \| **Class** \| **Tree** \| **Deprecated** \| **Index** \| **Help** \| **Weka's home** | |  |
| **PREV CLASS**   **NEXT CLASS** | **FRAMES**    **NO FRAMES**     **All Classes** |  |
| SUMMARY: NESTED \| FIELD \| CONSTR \| METHOD | DETAIL: FIELD \| CONSTR \| METHOD |  |


---
